# A Subscapularis-Sparing Modification of the Deltopectoral Approach for Facilitated Glenoid Exposure in Reverse Shoulder Arthroplasty: A Technical Note

**DOI:** 10.3390/jcm15082985

**Published:** 2026-04-14

**Authors:** Toru Ichiseki, Shusuke Ueda, Daisuke Soma, Keika Yasumoto, Ayumi Kaneuji, Norio Kawahara

**Affiliations:** 1 Department of Orthopaedic Surgery, Kanazawa Medical University, Daigaku 1-1, Uchinada-machi, Kahoku-gun 920-0293, Japan; adeu221@kanazawa-med.ac.jp (S.U.); ds0924@kanazawa-med.ac.jp (D.S.); kaneuji@kanazawa-med.ac.jp (A.K.);; 2 Division of Translational Research, Department of Life Science, Medical Research Institute, Kanazawa Medical University, Daigaku 1-1, Uchinada-machi, Kahoku-gun 920-0293, Japan

**Keywords:** reverse shoulder arthroplasty, deltopectoral approach, subscapularis preservation, glenoid exposure, surgical technique, shoulder arthroplasty approach

## Abstract

**Background:** In reverse shoulder arthroplasty (RSA), preservation of the subscapularis (SSC) has gained attention because of its biomechanical and functional significance. However, when SSC preservation is attempted using the conventional deltopectoral (DP) approach, glenoid visualization and instrument access may be limited. The purpose of this Technical Note is to describe a modified deltopectoral exposure technique, hereafter referred to as the Margin-Shifted, Yawing, Subscapularis-Sparing, and Transitioned Lateralized Deltopectoral (MYST–LDP) approach, and to assess its feasibility in primary RSA. **Methods:** The MYST–LDP approach incorporates a laterally shifted incision apex, a proximally oriented curved arc (“yawing”) toward the acromion, complete preservation of the subscapularis, and a distally transitioned limb aligned with the anterior humeral axis. We describe the surgical technique and our initial experience in three consecutive primary RSA cases performed using an inlay implant system to minimize humeral lateralization and allow focused assessment of exposure geometry. **Results:** In all cases, the SSC was preserved without conversion to a standard DP exposure. Adequate glenoid visualization was achieved using three retractors without excessive soft-tissue tension, allowing controlled glenoid preparation and component implantation without additional exposure or conversion. No approach-related intraoperative complications were observed. **Conclusions:** The MYST–LDP approach is a feasible modification of the deltopectoral exposure that preserves both SSC and the deltoid while facilitating glenoid visualization and instrument alignment. This technique represents an ergonomic and tissue-preserving option within the familiar DP framework for surgeons performing SSC-preserving RSA. Further comparative and quantitative studies are warranted to determine its clinical value.

## 1. Introduction

The deltopectoral (DP) approach remains the most widely used anterior approach for shoulder arthroplasty because of its clearly defined anatomical landmarks, extensile nature, and favorable safety profile. In reverse shoulder arthroplasty (RSA), the DP approach provides reliable access to both the humeral and glenoid sides and allows seamless extension of exposure when required, such as in cases of severe deformity, unexpected bone loss, or intraoperative complications. For these reasons, the DP approach continues to serve as the standard exposure for many shoulder surgeons worldwide and forms the foundation of most contemporary RSA techniques.

In recent years, increasing attention has been directed toward preservation of the subscapularis (SSC) during RSA. The SSC plays an important role in internal rotation strength, anterior shoulder stability, and maintenance of humeral head centering. Clinically, several studies have reported that preservation or repair of the subscapularis following RSA may be associated with improvement in internal rotation function and anterior stability, even when overall outcome scores are comparable [1,2,3]. These clinical observations are supported by biomechanical evidence, which suggests that subscapularis repair alters biomechanical loading conditions and contributes to joint stability and force transmission across the shoulder [4].

However, conventional shoulder arthroplasty techniques involving subscapularis detachment and subsequent repair raise concerns regarding the consistency of tendon healing. Ultrasound- and MRI-based imaging studies, as well as investigations using clinical examination, have reported postoperative subscapularis insufficiency or repair failure in approximately 20–50% of cases following shoulder arthroplasty, predominantly in the setting of anatomic total shoulder arthroplasty, with reported rates varying according to the assessment modality used [5,6,7]. These findings highlight the potential limitations of subscapularis repair from both biological and mechanical perspectives.

Furthermore, in the reverse shoulder arthroplasty setting, imaging-based evaluations focusing specifically on RSA have demonstrated that the structural integrity of the repaired subscapularis is variable, with reported healing or intact tendon rates generally ranging from approximately 30% to 70%, depending on implant design, assessment criteria, and imaging modality [8,9,10]. Thus, despite attempted repair, a substantial proportion of patients exhibit incomplete healing or postoperative insufficiency of the subscapularis.

Taken together, these observations support the concept that preservation of an intact subscapularis, when technically feasible, may represent a more biologically and functionally favorable strategy compared with routine detachment and repair in reverse shoulder arthroplasty.

In response, several SSC-sparing modifications of the DP approach have been reported [11,12]. These techniques demonstrate that SSC preservation through the DP interval is feasible and may facilitate early mobilization. However, most reported techniques retain a relatively medial and linear skin incision, and therefore, structural limitations related to visualization of the superior and posterosuperior glenoid, available working space, and instrument maneuverability may persist, particularly when attempting to optimize baseplate positioning and superior screw trajectory.

Accurate baseplate positioning, appropriate inferior tilt, and secure fixation are essential determinants of implant longevity and avoidance of complications such as instability or scapular notching [13,14]. Achieving these goals requires sufficient visualization and a favorable instrument trajectory toward the superior glenoid. Restricted visualization and constrained working corridors may increase technical demands, prolong operative time, and reduce reproducibility, especially in anatomically challenging cases or during early surgical experience.

Importantly, these limitations are not solely attributable to deep soft-tissue constraints, but are also influenced by the geometry of the skin incision itself, which determines the initial visual axis and working corridor toward the glenoid.

Based on these considerations, there remains a need for a surgical approach that preserves both the subscapularis and the deltoid, maintains the safety and familiarity of the deltopectoral interval, and overcomes the structural limitations related to glenoid visualization. We therefore developed a modified deltopectoral exposure technique, hereafter referred to as the Margin-Shifted, Yawing, Subscapularis-Sparing, and Transitioned Lateralized Deltopectoral (MYST–LDP) approach. Rather than introducing a new surgical plane, this technique focuses on structured modification of skin incision geometry to improve alignment between the visual axis and the instrument trajectory, while preserving the conventional DP workflow.

The purpose of this Technical Note is to describe the MYST–LDP approach in detail and to evaluate its initial technical feasibility in primary RSA.

## 2. Surgical Technique

### 2.1. Patient Positioning

The MYST–LDP approach can be performed on a standard operating table without the need for a dedicated beach-chair system. The patient is placed in the supine position with the torso elevated approximately 20°. A small pad is placed beneath the scapula to allow the scapular body to float anteriorly. The trunk is slightly shifted toward the operative side to secure adequate working space for upper-extremity extension. The arm is supported by a standard arm positioner or an assistant. During glenoid preparation, gentle rotation of the torso toward the contralateral side further enhances glenoid visualization. This simple setup allows the procedure to be performed even in facilities without specialized beach-chair equipment.

### 2.2. Skin Incision Design and Rationale

The defining feature of the MYST–LDP approach is a structured modification of the conventional DP skin incision, designed to optimize the visual axis and working corridor toward the glenoid without altering the underlying surgical interval.

The apex of the incision is shifted approximately 1.5 cm lateral to the coracoid process (margin shift). This lateralization repositions the skin window closer to the glenoid and reduces interference from medial soft tissues, including the conjoint tendon, pectoralis major, and coracobrachialis. The degree of lateralization is not intended as a precise measurement but rather as a practical guideline that can be adjusted according to patient habitus and surgeon preference. A lateral shift of approximately 1.5 cm was selected as an initial reference because it allows improved access while preserving straightforward identification of the deltopectoral interval and permitting seamless conversion to a standard DP approach if required.

From this lateralized apex, the incision is extended proximally and distally in two distinct components. Proximally, a smooth curved incision measuring approximately 2–3 cm is directed obliquely toward the lateral acromion (yawing arc). In this Technical Note, the term “yawing” is used purely as a descriptive analogy to indicate controlled reorientation of the proximal incision, rather than implying any formal biomechanical or kinematic construct. This orientation aligns the skin margin with the direction of retractor traction during glenoid exposure, reducing skin-edge interference and reactive counterpressure. As a result, retractors can be positioned more stably with less soft-tissue tension, facilitating access to the superior and posterosuperior glenoid.

Distally, the incision is extended longitudinally for approximately 6–8 cm along the anterior midline of the humeral shaft (transitioned DP limb). This distal configuration minimizes skin impingement during humeral manipulation and provides a more linear and ergonomic working corridor for canal preparation, broaching, and stem insertion, while preserving the familiar workflow of the traditional DP approach.

**Figure 1:**
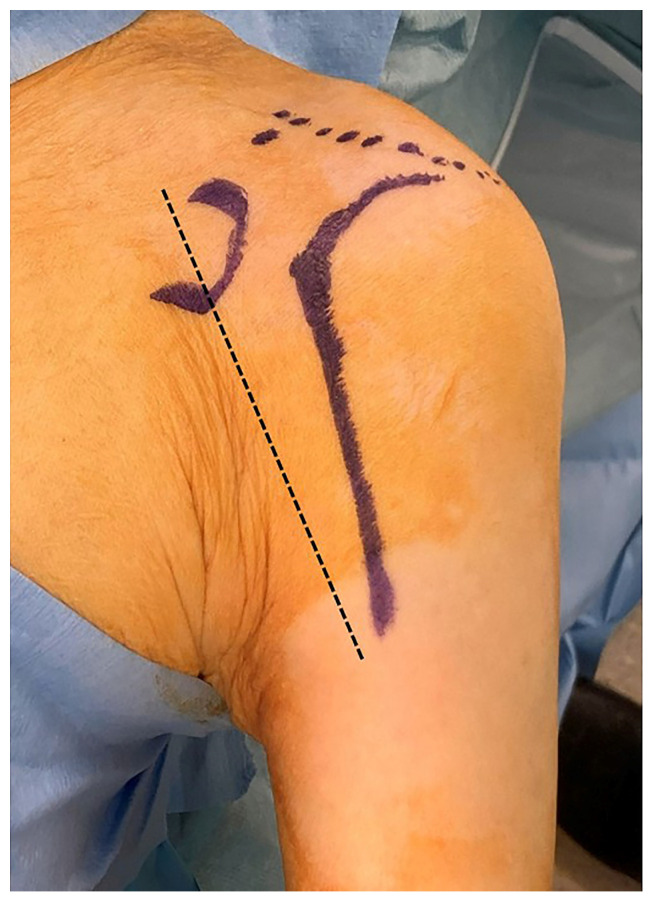
Skin incision design of the Margin-Shifted, Yawing, Subscapularis-Sparing, and Transitioned Lateralized Deltopectoral (MYST–LDP) approach. The solid line illustrates a laterally shifted incision apex with a proximally oriented curved (“yawing”) arc toward the acromion and a distally transitioned longitudinal limb aligned with the anterior humeral axis. The dashed line represents the conventional deltopectoral (DP) skin incision for comparison.

Importantly, this modification alters only the skin incision geometry and does not introduce any new surgical planes or modify the deltopectoral interval itself. All conventional anatomical landmarks and exposure principles are preserved, allowing unrestricted intraoperative conversion to a standard DP approach when necessary (Figure 1).

### 2.3. Deltopectoral Interval Exposure

The cephalic vein is preserved and retracted either laterally with the deltoid or medially with the pectoralis major, according to local anatomy and surgeon preference. Because the skin incision is lateralized, compression and traction on the anterior deltoid are reduced compared with the conventional DP approach, which may decrease soft-tissue strain during glenoid exposure and instrumentation.

### 2.4. Management of the Long Head of the Biceps

The long head of the biceps tendon is identified within the bicipital groove and treated with soft-tissue tenodesis at the level of the pectoralis major insertion. Despite the lateralized incision, identification and management of the tendon follow the familiar DP workflow, facilitating adoption of the technique by surgeons accustomed to the standard approach.

### 2.5. Preservation of the Subscapularis

The subscapularis (SSC) is completely preserved without tenotomy or soft-tissue detachment. External rotation of the humerus brings the lesser tuberosity anteriorly, allowing sufficient exposure with only gentle superior and inferior retraction, without detachment of the tendon. Preservation of the SSC eliminates the need for tendon repair and may facilitate earlier initiation of postoperative range-of-motion exercises, depending on surgeon preference and postoperative protocol.

### 2.6. Humeral Preparation

**Figure 2:**
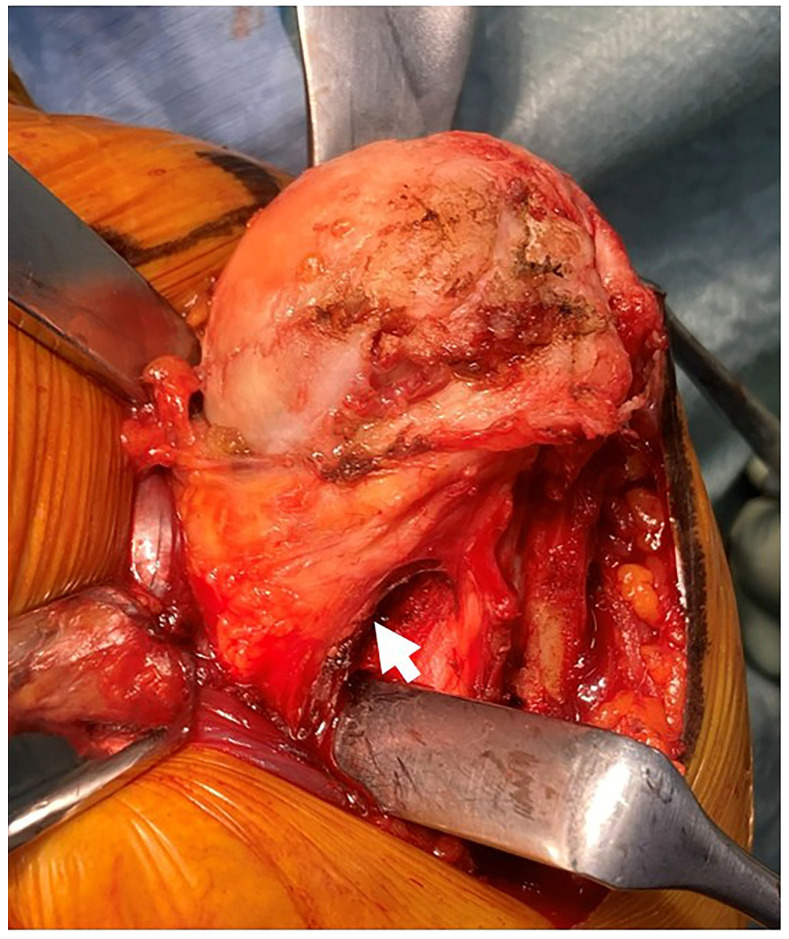
Humeral head exposure with preserved subscapularis. Intraoperative view demonstrating humeral head exposure achieved through the MYST–LDP approach with complete preservation of the subscapularis, without tenotomy or tendon detachment. The preserved subscapularis tendon is indicated by a white arrow.

With the subscapularis preserved, the humeral head is gently dislocated anteriorly (Figure 2), and humeral head resection is performed. Even in the presence of inferior humeral osteophytes, these can be safely removed under direct visualization with minimal release of medial soft tissues and gentle superior traction on the preserved SSC. Following humeral head removal, exposure of the glenoid becomes substantially easier.

The proximally yawed curved skin incision directed toward the acromion reduces skin interference around the humeral head and proximal humerus, allowing smooth and controlled exposure during humeral head manipulation.

Furthermore, linearization of the distal limb of the incision reduces skin and soft-tissue impingement during canal preparation and stem insertion. Compared with the conventional DP approach, this configuration minimizes excessive soft-tissue tension and provides a more natural working corridor with improved instrument entry angles during intramedullary reaming, broaching, and stem insertion.

### 2.7. Glenoid Exposure and Component Implantation

In this approach, adequate glenoid exposure is typically obtained using three retractors placed anteriorly, inferiorly, and posteriorly, without requiring excessive assistant-dependent traction. Owing to the lateralized and proximally yawed incision, posterior retractor placement does not cause significant skin-edge impingement, allowing stable retraction without excessive soft-tissue tension.

When additional superior visualization is required, a superior retractor may be placed beneath the undersurface of the acromion to provide direct access to the glenoid surface. This configuration improves alignment between the visual axis and the operative corridor, thereby reducing instrument impingement within the wound and facilitating controlled component implantation.

**Figure 3:**
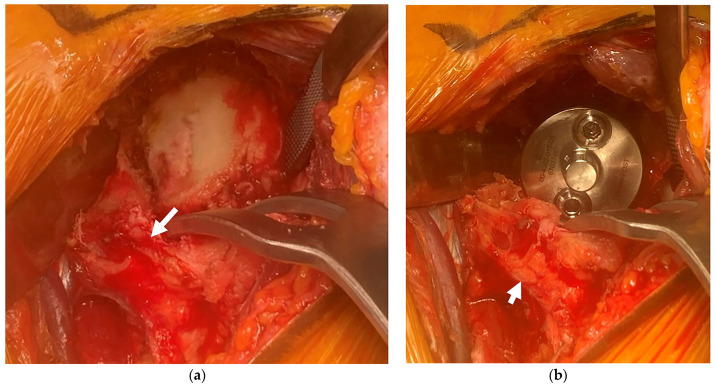
Glenoid exposure and baseplate placement through the MYST–LDP approach. (**a**) Intraoperative view demonstrating adequate glenoid exposure achieved through the MYST–LDP approach using three retractors. (**b**) Glenoid baseplate placement performed through the same exposure. The preserved subscapularis tendon is indicated by a white arrow.

As a result, glenoid reaming, baseplate positioning, and screw fixation can be performed safely and in a controlled manner (Figure 3).

### 2.8. Humeral Preparation, Stem Insertion, and Reduction

The transitioned DP limb aligns centrally along the anterior aspect of the humerus, and the laterally yawed skin incision minimizes soft-tissue impingement around the proximal humerus. This configuration allows humeral preparation to be performed slightly lateral to the standard DP axis, providing a more direct pathway for canal reaming, broaching, and stem insertion with fewer mechanical constraints compared with the traditional DP approach.

**Figure 4:**
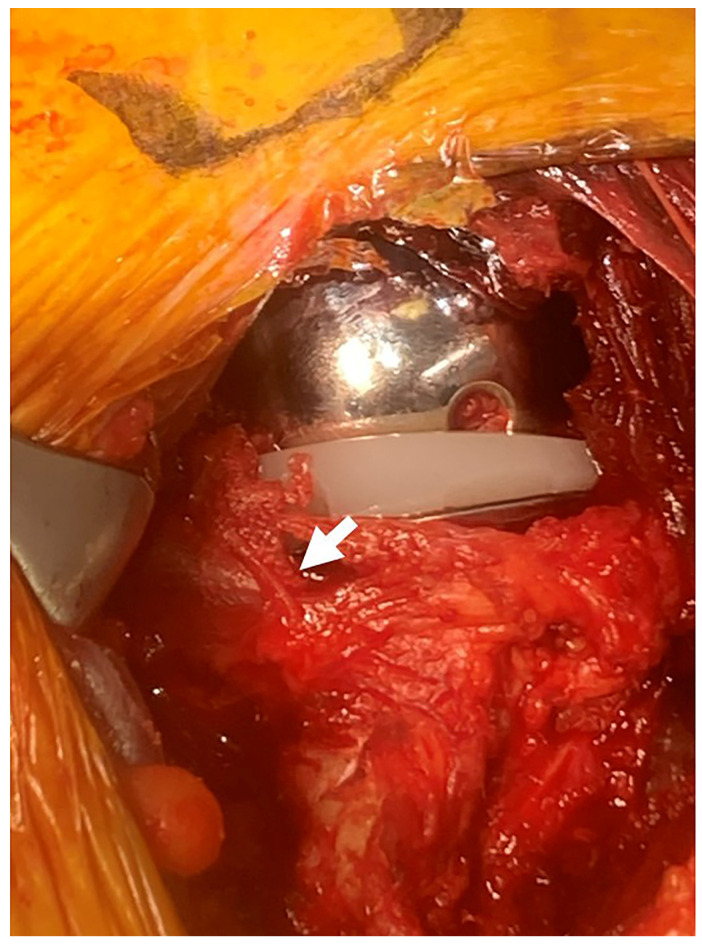
Final intraoperative view after reverse shoulder arthroplasty. Intraoperative view after completion of reverse shoulder arthroplasty through the MYST–LDP approach, demonstrating preservation of the subscapularis through the same exposure. The preserved subscapularis tendon is indicated by a white arrow.

Following final stem implantation, the components are reduced in a routine manner (Figure 4).

### 2.9. Closure and Postoperative Rehabilitation

The wound is closed in multiple layers, and tendon repair is unnecessary because the SSC is not detached. Postoperatively, passive and active-assisted range-of-motion exercises are initiated early, with gradual progression to active motion as tolerated by pain. In our protocol, active-assisted and active elevation exercises begin on postoperative day 1, and sling use is limited to short-term pain control during the early postoperative period.

### 2.10. Summary of Surgical Workflow

This approach maintains the familiar DP workflow, requires no additional equipment, and preserves all major soft-tissue structures, including the SSC and deltoid, while improving glenoid access and surgical ergonomics.

## 3. Initial Technical Experience

As an initial proof-of-concept series, the MYST–LDP approach was applied in three consecutive cases of primary reverse shoulder arthroplasty performed using an inlay implant system to minimize humeral lateralization. In all cases, preservation of the subscapularis (SSC) was achieved without conversion to a standard DP approach. Adequate glenoid visualization was consistently obtained using three retractors, allowing controlled glenoid preparation and component implantation without the need for additional exposure or extensile soft-tissue release. The surgical procedures were completed without the need for conversion to a standard extensile approach.

No approach-related intraoperative complications were observed, including deltoid violation, cephalic vein injury, neurovascular injury, intraoperative fractures caused by excessive traction or manipulation, or the need for conversion to an extensile approach.

The small number of cases reflects the intent of this report as a pure technical feasibility assessment rather than an outcome study. The primary aim was to confirm that the proposed incision geometry could reproducibly allow SSC preservation while providing sufficient glenoid exposure for safe and controlled component implantation, without the need for conversion or additional releases. Accordingly, no conclusions regarding clinical or radiographic outcomes or superiority over other surgical approaches can be drawn from this limited series, and further studies are warranted to evaluate the clinical relevance of this technique.

## 4. Discussion

The MYST–LDP approach introduces a conceptual emphasis on incision design beyond previously described SSC-sparing deltopectoral techniques, addressing geometric constraints inherent to the traditional DP exposure rather than focusing solely on tendon preservation.

To our knowledge, previously reported SSC-sparing DP approaches have not specifically emphasized structured geometric modification of skin incision design to alter the visual axis.

Previous SSC-sparing DP approaches have demonstrated that preservation of the subscapularis through the deltopectoral interval is technically feasible. However, most of these techniques adopted predominantly medial and linear skin incisions and did not specifically aim to actively adjust the directionality of the visual axis or instrument trajectory through incision geometry itself. In contrast, the MYST–LDP technique intentionally reconfigures the spatial relationship between the incision apex, exposure angle, and operative corridor toward the glenoid. By laterally shifting the incision apex and incorporating a proximally oriented yawing arc, the approach improves alignment between the visual axis and the instrument trajectory toward the glenoid. This geometric realignment may reduce reliance on aggressive soft-tissue retraction, enhance instrument ergonomics, and facilitate more reproducible component positioning in primary reverse shoulder arthroplasty.

From a conceptual standpoint, the MYST–LDP approach represents an interface-level modification rather than a deep anatomical alteration.

Because reverse shoulder arthroplasty is a deltoid-dependent procedure, preservation of deltoid integrity remains an important consideration [15]. Anterosuperior and lateral approaches may improve glenoid exposure in selected cases [16,17,18]. However, these approaches may involve deltoid splitting or limited detachment and have been associated with concerns regarding deltoid integrity and neurologic risk [16].

In contrast, the present MYST–LDP approach seeks to improve glenoid visualization while preserving both the deltoid and the subscapularis within the familiar deltopectoral framework. In our experience, the MYST–LDP approach may be particularly well suited for primary reverse shoulder arthroplasty cases in which preservation of the subscapularis is feasible and adequate glenoid exposure is anticipated through the deltopectoral interval.

The purpose of this technique is not to mandate subscapularis preservation in all cases, but rather to provide a reproducible option when preservation is considered beneficial by the surgeon. Although the clinical impact of subscapularis repair remains controversial, concerns such as postoperative re-tear and its potential relationship with joint stability have been reported. Therefore, preserving the subscapularis may be desirable when feasible.

Conversely, relative limitations of this approach may include cases with severe anterior soft-tissue contracture or substantial scarring resulting from prior surgery. In addition, in cases with advanced glenoid bone loss requiring extensile exposure, conversion to a standard deltopectoral approach may be more appropriate.

Importantly, the goal of the present technique is not to replace established surgical approaches. Both anterosuperior and lateral approaches remain valuable strategies in RSA, particularly in experienced hands. Rather, the MYST–LDP approach is intended to offer an additional soft tissue-preserving option within the familiar DP framework. Because this approach remains fully compatible with the standard DP workflow, seamless intraoperative conversion to a conventional DP exposure is possible if required, minimizing technical risk and maintaining surgical familiarity. In addition, this approach is not expected to compromise future revision surgery, as conversion to a standard deltopectoral exposure remains feasible. This continuity may also facilitate adoption and shorten the learning curve for surgeons transitioning toward SSC-preserving RSA strategies.

In this initial experience, consistent glenoid visualization was achieved using three retractors. Although this report was not designed to evaluate clinical outcomes, radiographic accuracy, or operative efficiency, the improved exposure pathway may theoretically contribute to more reproducible baseplate positioning, optimized screw trajectory, and protection of surrounding soft tissues. Glenoid preparation was completed without the need for accessory portals or extensile soft-tissue release in all cases.

Additional intraoperative images from the remaining cases demonstrating glenoid exposure with subscapularis preservation are provided as Supplementary Materials to support the reproducibility of the technique (Supplementary Figure S1).

Furthermore, the degree of lateralization of the incision in this technique is modest and does not compromise exposure of the coracoid base. Therefore, the approach is considered compatible with navigation systems requiring coracoid-based tracker placement.

These potential advantages may be particularly relevant in anatomically complex primary cases in which sufficient exposure can still be achieved through the deltopectoral interval.

This report has several limitations. The number of cases is small, and no quantitative clinical or radiographic outcome measures were assessed. All procedures were performed using an inlay implant design, and the applicability of this approach to onlay systems remains to be determined. This choice was made solely for exposure assessment and does not imply a limitation of the technique to a specific implant design. In addition, no direct comparison was made with previously reported SSC-sparing deltopectoral techniques; therefore, further comparative studies are required to determine whether the geometric advantages described translate into improved radiographic accuracy or clinical outcomes.

Nevertheless, the primary purpose of this Technical Note is to introduce a reproducible and technically intuitive modification of the deltopectoral approach that provides a conceptual framework for achieving both SSC preservation and improved glenoid visualization. These observations support the initial technical feasibility that formed the purpose of this report.

In summary, the MYST–LDP approach represents a deltoid- and SSC-preserving modification of the conventional DP exposure that improves glenoid visualization and instrument alignment while maintaining surgical familiarity. This incision-based modification can be readily adopted by surgeons familiar with the standard deltopectoral approach without additional equipment or specialized training. The technique is intended as an additional ergonomic option rather than a replacement for existing RSA exposures, and may allow surgeons to pursue SSC-preserving RSA within an easily adoptable, DP-based surgical strategy. Future studies assessing comparative exposure metrics, implant positioning accuracy, and functional outcomes are warranted to define the full clinical value of this approach.

## 5. Conclusions

The MYST–LDP approach is a technically feasible modification of the deltopectoral exposure that preserves both the SSC and the deltoid while facilitating glenoid visualization. This technique may provide an ergonomic and tissue-preserving alternative within the familiar DP framework and enable surgeons to perform SSC-preserving RSA through a reproducible workflow. Further quantitative and comparative studies are warranted to determine its clinical impact and evaluate long-term outcomes.

## Data Availability

The data presented in this study are available on request from the corresponding author due to privacy considerations.

## Supplementary Materials

The following supporting information can be downloaded at: https://www.mdpi.com/article/10.3390/jcm15082985/s1, Supplementary Figure S1: lntraoperative images from two additional cases demonstrating adequate glenoid exposure with preservation of the subscapularis. The white arrows indicate the preserved subscapularis tendon. (a) Case 2; (b) Case 3.

## Abbreviations

The following abbreviations are used in this manuscript:

RSA	Reverse shoulder arthroplasty
DP	Deltopectoral
SSC	Subscapularis
MYST–LDP	Margin-Shifted, Yawing, Subscapularis-Sparing, Transitioned Lateralized Deltopectoral
