# Supplementary material for: A Subscapularis-Sparing Modification of the Deltopectoral Approach for Facilitated Glenoid Exposure in Reverse Shoulder Arthroplasty: A Technical Note
Source: J Clin Med. 2026 Apr 14;15(8):2985. doi: 10.3390/jcm15082985 (PMC13116733; doi:10.3390/jcm15082985)
Supplement: Supplementary file 1 [file jcm-15-02985-s001.zip › jcm-4212402-supplementary.pdf]

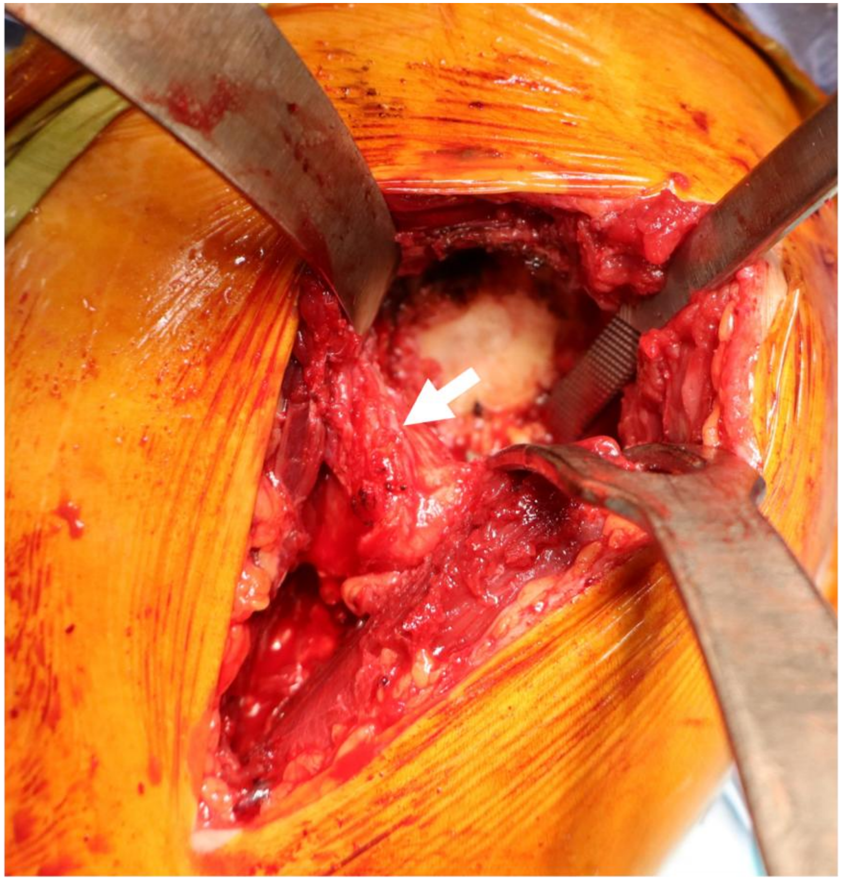

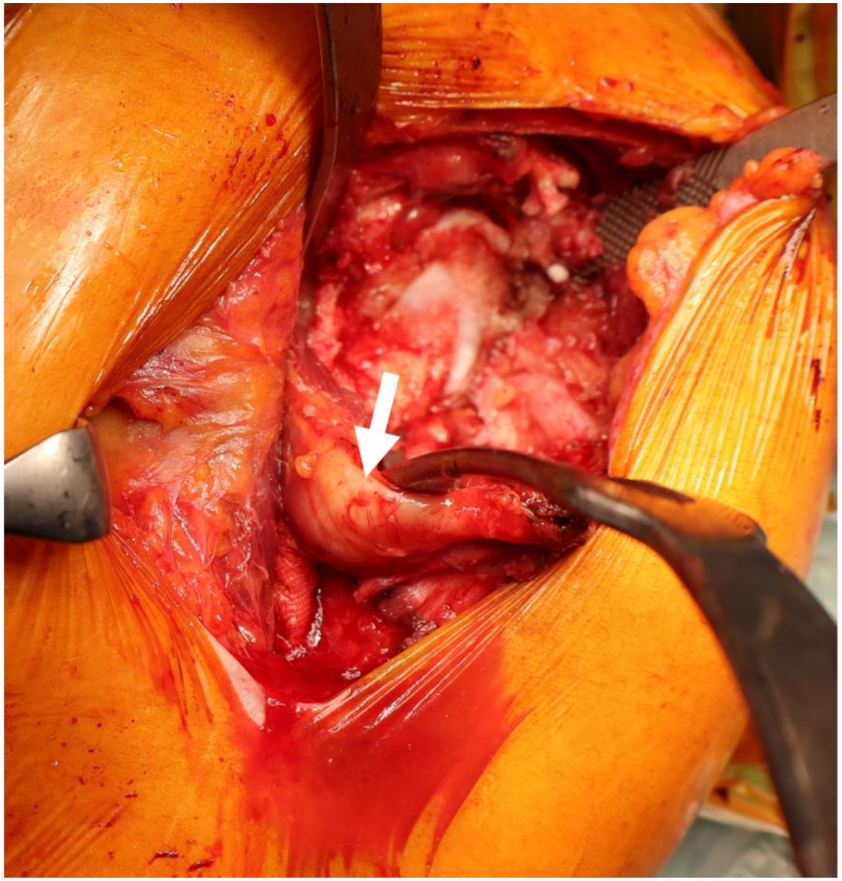

(a) Case 2

(b) Case 3

Supplementary Figure S1.
Intraoperative images from two additional cases demonstrating adequate glenoid exposure with preservation of the subscapularis. The white arrows indicate the preserved subscapularis tendon. (a) Case 2; (b) Case 3.
